# Counsel's Opinion

**Published:** 1886-12-11

**Authors:** Harry T. Eve

**Affiliations:** 4, New Square, Lincoln's Inn


### COUNSEL'S OPINION.

I have perused the papers and correspondence herein, and in my opinion the course adopted by the proprietors of *The Globe* is a good ground for an action for damages against them.

In considering the various points raised, it must be borne in mind that the article was, at the time it was obtained by *The Globe*, unpublished; that the right of the author or proprietor therein was therefore a common law right of property as distinct from a statutory copyright; and that in determining the nature of the wrong done and the remedy to be adopted, it is necessary to regard the question as an interference with the ordinary rights of property, and not as an interference with a special property so made by Act of Parliament.

Now, in the first place, it is settled law that every product of mental labour which has been embodied in writing, or some other material form, becomes the exclusive property of the author, and that without his consent the law will not allow any other person to publish it, and that this property and the consequent power to protect it exists independent of Statute. This law was emphasised with great force in the judgments delivered in *Millar v. Taylor*, decided in 1769, and reported in Volume 4 of Burrow's Reports:—

"It is settled," says Aston, J., at page 2340, "and admitted, and is not now controvertible, but that literary compositions in their original state and the incorporeal right of the publication of them are the private and exclusive property of the author, and that they may ever be retained so, and that if they are ravished from him, before publication, trover or trespass lies."

Again per Willes, J., at page 2331:—

"It is clear that there is a time when, without any positive statute, an author has property in the copy of his own work in the legal sense of the word."

In the same case instances were cited by the Court, of what would amount to a violation of this right of property, and as they are somewhat analogous to the present case, I extract the following:—

"Suppose," says Willes, J., at page 2330, "a man with or without leave to peruse a manuscript work transcribes and publishes it—it is not within the Act of Queen Anne—it is not larceny—it is not trespass—it is not an indictable offence (the physical property of the author, the original manuscript remains), but it is a gross violation of a valuable right."

"Suppose," says Willes, J., again on the same page, "the original or a manuscript was given or lent to a man to read for his own use, and he published it, it would be a violation of the author's common law right to the copy. This never was doubted, and has often been determined."

At page 2,360, Yates, J., put almost the present case. He says :—

"If a stranger had taken his (the plaintiff's) manuscript from him, or had surreptitiously obtained a copy of his work and printed it before him, he might complain of injustice. In that case it would be the highest injustice."

The case from which the foregoing are extracts is still regarded as the leading case on the question of an author's property in his unpublished productions, and was followed, and its application somewhat extended, in the more recent case of *Prince Albert v. Strange* (1 De Gex and Smale, 652). It is, therefore, as I have already stated, settled law that the author of unpublished manuscripts has a right, independent of statute, to the exclusive use of them, and can prevent the publication of them by anyone else.

This being so, it is clear that Lord Salisbury could maintain an action against *The Globe* for the infringement of his right of first publication ; and this conclusion gives rise to the second question, whether the proprietors of THE HOSPITAL can equally maintain such an action. In my opinion they can, and for this reason—that, once admitted that the author's right of first publication is property in the legal sense of the term, it follows, as an incident, that it can be transferred by the author, otherwise the author, to preserve his right, would necessarily have himself to make the first publication. Moreover, as regards unpublished matter, there is no statutory enactment as to the assignment of the copyright; and it appears to me, therefore, that such assignment can be proved by parol evidence, and is in no way affected by the decision in *Leyland v. Stewart* (L. R. 4 Ch. Div. 419) and the cases therein referred to.

The last question is as to the nature of the action. I think the writ should be issued in the Queen's Bench Division by the proprietors of THE HOSPITAL, or other the person or persons to whom the property was transferred by Lord Salisbury, as plaintiffs, against the registered proprietors of *The Globe*, as defendants, and that the indorsement should be "for damages for the infringement of the plaintiff's copyright in an unpublished manuscript, to wit, an article by the Right Hon. Lord Salisbury, K.C.B., etc., etc., entitled, etc., etc., and for costs.

HARRY T. EVE,
4, New Square, Lincoln's Inn,
4th Dec., 1886.

